# Milk and Milk-Contracts
*Previous articles appeared on May 3, 10, 17, 24, 31, and June 14.


**Published:** 1913-06-28

**Authors:** Everitt E. Norton

**Affiliations:** Medical Superintendent of Isleworth Infirmary.


### MILK AND MILK-CONTRACTS.*

By EVERITT E. NORTON, M.D., D.P.H., etc., Medical Superintendent of Isleworth Infirmary.

### VII.—The Rational Basis of a Milk-Contract.

In what terms, under existing conditions, can a milk-contract be best framed? The object to be attained, and it is one which is attainable, is to secure the delivery in London of clean milk in a chilled state; to obtain such milk is the business of the purchaser. The essential requirements are (i) the exclusion as a source of supply of cows suffering from tuberculous disease of the udder, (ii) cleanliness in farm methods, and (iii) efficient and sustained cooling of the milk.

It may be said that the time is rapidly approaching when, as the result of legal enactments and the activity of public authorities, all that is necessary will be done. So happy a state of things may be hoped for, but its advent must not be depended upon; those whose duty it is to provide milk for others, especially for large numbers of sick persons, should not be content to weakly depend upon so vague an anticipation.

### Importance of Definite and Reasonable Terms.

It is futile to frame regulations which will be disregarded or to threaten inspections which will never be made. I have in a previous article mentioned a contract-form which stipulates that the milk to be supplied "*should* be cooled (the italics are mine) to a temperature of 50° before delivery," but which goes no further than the expression of that opinion! A contract must be worded in definite and reasonable terms and must protect the rights of the purchaser in such a manner as to give a fair prospect of its honest fulfilment. "Aggressive programmes of milk reform," which, as Dr. Eastwood says, "have not succeeded in America and are not to be recommended" from the public health point of view, certainly have no place in the consideration of institution milk-contracts.

### Supervision by Skilled Inspectors.

It is, at least at present, absolutely necessary that provision be made for the supervision, by skilled inspectors appointed by and acting on behalf of the purchaser, of the manner in which in its smallest details a contract is carried out. It is advisable that such inspectors should be independent and responsible persons and, for obvious reasons, neither too local to the farms nor resident at a great distance from them.

I am intentionally making no suggestions as to the general conditions of contracts which, being rather legal than medical considerations, do not fall within the scope of my subject. Consequently I say nothing concerning provisions as to penalties or forfeits, the settlement of disputes, arbitration, guarantees, bonds or sureties, means of enforcing fulfilment, power to purchase in default, the disposal of rejected goods, transfer of contracts, bribery, or requirements as to notices. A contract should, of course, include provisions as to quantities, times of delivery, and collections of empties. It might, possibly, rarely occur that a contractor would (through no default of his own) be obliged to temporarily suspend deliveries at the request of the purchaser; in such a case he should, of course, be at least indemnified as regards any penalty or forfeit.

In suggesting the following conditions as applicable to a milk-contract I realise that some omissions or modifications might be necessary to adapt them to individual cases. For the sake of simplicity the "purchaser" and the "contractor" are throughout referred to.

### The Standard of Quality.

The contract should specify that the milk is to be the pure raw milk of healthy cows, without the fore-milk but containing all the strippings and all the natural milk-fat, perfectly clean and sweet on delivery; free from added water, sugar, preservatives, chemicals, colouring-matter, any foreign matter or adulteration whatsoever, and from any unnatural odour or taste, whether resulting from the feeding of the cows or from any other cause; untreated by heat and (unless with the sanction in writing of the purchaser) not passed through any separator, through any centrifugal or other machine of any kind, or through pipes or tanks.

The milk must contain not less than 8.50 per cent. by weight of solids not fat; and of milk-fat, during the months of September to January inclusive, not less than 3.50, and during the months of February to August inclusive, not less than 3.25 per cent. by weight.

It must, on delivery, show a bacterial content never exceeding 500,000 organisms per cubic centimetre, and contain no sediment exceeding 40 vols. per million.

### Duties of the Contractor.

The contractor must see that the provisions of any acts, orders or by-laws applicable to his business, and to that of the farmers dealt with by him, are strictly complied with.

No cow must be used as a source of supply within sixty days before calving be due, nor until the tenth day after calving.

No cow affected by any disease of the udder must be used as a source of supply, nor must any cow known to be suffering from any form of tuberculous disease be so used.

Any cow found to be suffering from tuberculous disease of the udder must be, within twenty-four hours of being so found, removed from the herd and from the farm; and any cow found to be suffering from any other form of tuberculous disease must be, within seven days of being so found, similarly removed.

* Previous articles appeared on May 3, 10, 17, 24, 31, and June 14.

The milk of each cow must be removed, unmixed with that of any other cow, from the cowshed immediately after milking, and be at once efficiently strained through a strainer of approved construction.

The milk is to be afterwards, without delay, cooled by a method and apparatus to be approved, to a degree of temperature which shall not exceed one to be agreed upon, in consultation with an inspector acting for the purchaser.

The milk to be supplied should be obtained only from certain specified farms, as few in number as can conveniently furnish the required quantities, to be approved by, or on behalf of, the purchaser. The farms supplying should not be, at any time, more in number than the number agreed upon before the sealing of the contract. No milk from any farm other than those originally named should be substituted or delivered unless seven days' notice in writing has been previously sent to the purchaser and such farm has been accepted after due inspection.

Suggested Regulations for Delivery.

The milk should be delivered only in railway churns or cans of a pattern which has been submitted to, and approved by, the purchaser. The cans or railway churns must be proof against the admission of dust or rain, and have a lid so fitted that in the event of the contained milk splashing over or running out it cannot pass back into the churn; they must have no ventilating holes; those in use must be clean, sound, and in good condition. They should have engraved upon them the name and address of the farmer or of the contractor as may be required by the purchaser, and must in any case, upon every delivery, bear upon an engraved metal label the address of the farm from which the milk has been obtained, and the occupier's name. If necessary, for the purposes of a railway company or otherwise, the tare-weight of each churn should be engraved or stamped upon the outside. The churns must, upon every delivery, be locked or sealed at the farm in a manner approved by the purchaser, and must not be opened during transit except by an inspector legally authorised to take samples of the milk. They must be delivered direct and unopened, by the most expeditious means and route, from the farm to the purchaser without any unavoidable delays whatever. Each delivery must be only of milk derived from the appropriate milkings last before the time appointed for delivery. The churns should be thoroughly washed before being returned to the contractor.

Facilities for Checking Fulfilment of Terms.

The contractor must afford to any authorised representative inspecting, or acting, on behalf of the purchaser all facilities for seeing that the terms of the contract can be, and are being, duly fulfilled; for obtaining any relevant information (including any statement of fact or opinion from public officers, inspectors, or others visiting the farms); for making any necessary examinations; and for taking any samples. He must similarly afford facilities for the inspection of all farm records, counterfoils, etc.

It will be necessary for the contractor, before the sealing of the contract, to submit for the approval of the purchaser or his representatives:—

(i) The addresses of the farms from some or all of which it is proposed to obtain the milk and the names of the occupiers;

(ii) the regulations to be enforced at the farms in order to ensure cleanliness and efficiency in the milking and canning processes and everything relating thereto;

(iii) the degree of temperature to which he proposes to undertake that the milk shall be cooled at the farms; and

(iv) a specimen railway churn or can, showing the size, pattern, construction, methods of marking, and means of locking or sealing, such as it is proposed to use for delivery.

The contractor should supply the purchaser, week by week, within seventy-two hours of the completion of each week, with a return showing the quantities of milk furnished at each delivery from each of the approved farms. He should at the same time sign a declaration that, to the best of his knowledge, belief, and information, the terms of the contract are being in all respects complied with, the farm regulations are being enforced, the sanitary conditions remain good, and that no occurrence requiring to be reported by him remains unreported.

